# Clinical score for early diagnosis of myotonic dystrophy type 2

**DOI:** 10.1007/s10072-022-06507-9

**Published:** 2022-11-19

**Authors:** Vukan Ivanovic, Stojan Peric, Jovan Pesovic, Radoje Tubic, Ivo Bozovic, Ivana Petrovic Djordjevic, Dusanka Savic-Pavicevic, Giovanni Meola, Vidosava Rakocevic-Stojanovic

**Affiliations:** 1 University of Belgrade – Faculty of Medicine, University Clinical Center of Serbia – Neurology Clinic, Dr. Subotic Street, 11 000, Belgrade, Serbia; 2 University of Belgrade – Faculty of Biology, Center for Human Molecular Genetics, Belgrade, Serbia; 3 Institute of Oncology and Radiology of Serbia, Belgrade, Serbia; 4 University of Belgrade – Faculty of Medicine, University Clinical Center of Serbia – Cardiology Clinic, Belgrade, Serbia; 5 Department of Neurorehabilitation Sciences - Casa Di Cura del Policlinico, Department of Biomedical Sciences for Health, University of Milan, Milan, Italy

**Keywords:** Myotonic dystrophy type 2 (DM2), Clinical score, Cataracts, Myotonia, Tremor

## Abstract

**Introduction:**

Myotonic dystrophy type 2 (DM2) is a rare, multisystemic, autosomal dominant disease with highly variable clinical presentation. DM2 is considered to be highly underdiagnosed.

**Objective:**

The aim of this study was to determine which symptoms, signs, and diagnostic findings in patients referred to neurological outpatient units are the most indicative to arouse suspicion of DM2. We tried to make a useful and easy-to-administer clinical scoring system for early diagnosis of DM2-DM2 early diagnosis score (DM2-EDS).

**Patients and methods:**

Two hundred ninety-one patients with a clinical suspicion of DM2 were included: 69 were genetically confirmed to have DM2, and 222 patients were DM2 negative. Relevant history, neurological, and paraclinical data were obtained from the electronic medical records.

**Results:**

The following parameters appeared as significant predictors of DM2 diagnosis: cataracts (beta = 0.410, *p* < 0.001), myotonia on needle EMG (beta = 0.298, *p* < 0.001), hand tremor (beta = 0.211, *p* = 0.001), positive family history (beta = 0.171, *p* = 0.012), and calf hypertrophy (beta = 0.120, *p* = 0.043). In the final DM2-EDS, based on the beta values, symptoms were associated with the following values: cataracts (present 3.4, absent 0), myotonia (present 2.5, absent 0), tremor (present 1.7, absent 0), family history (positive 1.4, negative 0), and calf hypertrophy (present 1.0, absent 0). A cut-off value on DM2-EDS of 3.25 of maximum 10 points had a sensitivity of 84% and specificity of 81% to diagnose DM2.

**Conclusion:**

Significant predictors of DM2 diagnosis in the neurology outpatient unit were identified. We made an easy-to-administer DM2-EDS score for early diagnosis of DM2.

**Supplementary information:**

The online version contains supplementary material available at 10.1007/s10072-022-06507-9.

## Introduction

Myotonic dystrophy type 2 (DM2) is a rare, multisystemic, late-onset, slowly progressive, and clinically highly variable autosomal dominant hereditary disorder, which is caused by an unstable CCTG expansion located in the intron 1 of the *CNBP* gene [1–3]. The main clinical characteristics of DM2 are slowly progressive proximal muscle weakness, myalgia, myotonia, and cataracts, but also cardiac, endocrine, and smooth muscle abnormalities are seen. Besides this, DM2 also affects the brain [4, 5]. DM2 has some of the most diverse disease-related symptoms among human disorders, which may be the main cause of the under-diagnosis of the disease [6]. The majority of DM2 symptoms are amenable to treatment which may improve patients’ quality of life and disease prognosis. DM2 usually starts in the third or fourth decade of life, i.e., in working age, which may pose a significant burden on society, not only on an individual and family [7]. According to the results of a population genetics study conducted in Finland, the frequency of *CNBP* gene mutation in the general population is significantly higher than the frequency of recognized cases of the disease, which can be caused by incomplete penetrance and variable expression of the mutated gene and/or insufficient recognition of mild disease symptoms by a general practitioner and neurologists [8].

To partially overcome the problem of DM2 under-diagnosis, we have previously performed genetic screening for DM2 in patients with presenile cataracts. Among 150 early-onset cataracts patients without any neurological symptoms, even 11 (7%) were genetically positive for DM2 [9]. Similarly, researchers in Finland conducted genetic screening for DM2 in 63 patients with fibromyalgia and found two (3%) cases of DM2 [10]. However, this finding was not confirmed in a Dutch study that showed no prevalence excess of DM2 in patients with suspected fibromyalgia [11]. It is of note that the most common first symptoms in the Serbian cohort of DM2 patients, besides lower limb weakness (37%) and handgrip myotonia (16%), were limb pain (10%), cataracts (8%), and even parkinsonism (3%) [12]. All these findings suggest that clinicians must be aware of high variability of DM2 clinical presentation, which would eventually lead to a better diagnosis of DM2. However, it remains unclear under which circumstances patients from neurological outpatients should be tested for DM2.

The aim of this study was to determine which symptoms, signs, and diagnostic findings in patients referred to neurologists are the most indicative to arouse suspicion of DM2. Based on these results, we tried to make a useful and easy-to-administer clinical scoring system for the early diagnosis of DM2.

## Patients and method

The study included all patients with a clinical suspicion of DM2 made by a neurologist in a tertiary university hospital. There were not any predefined criteria on which suspicion of DM2 was made by clinicians. Any neurologist from our hospital could send samples for DM2 testing if he had any suspicion of this disorder. In this way, we were able to assess many clinical and paraclinical features without any bias toward predefined signs and symptoms.

**Fig. 1:**
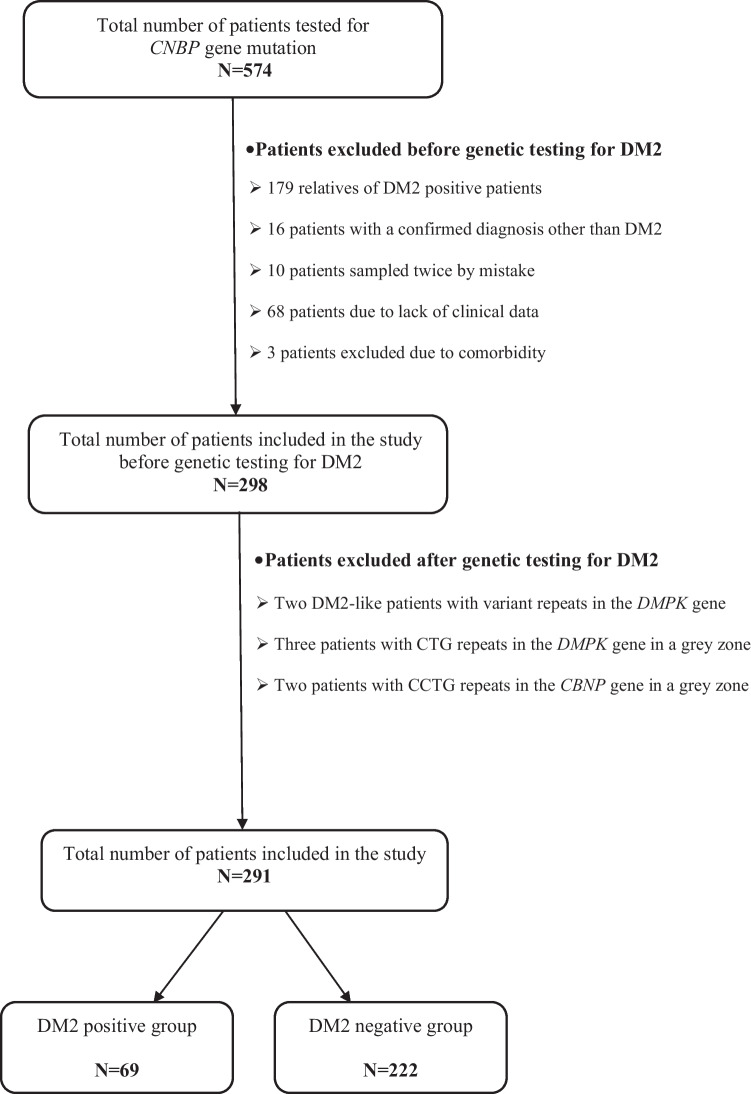
Flowchart showing included and excluded patients

Blood samples of these patients were referred to the Centre for Human Molecular Genetics at the Faculty of Biology, University of Belgrade for a repeat-primed polymerase chain reaction (RT-PCR) to detect CCTG repeat expansion in intron 1 of the *CNBP* gene [13]. From January 2013 (when a routine genetic analysis of DM2 was introduced in Serbia) until December 2019, 574 such patients were identified. To reduce selection bias, we excluded all relatives of DM2-positive patients (179 subjects). We further excluded 16 patients in whom diagnosis other than DM2 was confirmed before DM2 genetic testing was performed. After other diagnosis was made, their doctors decided to recall request for genetic analysis on DM2, and it was not performed at all. We also excluded ten patients whose blood was sampled twice by mistake. For 68 patients, clinical data were lacking since their blood samples were sent from other institutions. We also excluded three patients diagnosed with DM2 and another neurological disorder (double trouble) that could affect the interpretation of the clinical findings (myotonic dystrophy type 1, myasthenia gravis, and amyotrophic lateral sclerosis). After the genetic analysis was performed, we excluded additional seven patients: two patients with typical clinical presentation of DM2 that were genetically diagnosed as DM1 with variant repeats in the *DMPK* gene [14], three patients with CTG repeats in the *DMPK* gene in a grey zone (34–39 repeats), and two patients with CCTG repeats in the *CNBP* gene in a grey zone (35–50 repeats). The final number of patients included in the study was 291. Among these patients, 69 were genetically confirmed to have DM2 (DM2 + group) and 222 patients without CCTG expansion in the *CNBP* gene (DM2 − group) (Fig. 1).

By reviewing medical records, the following data were obtained: gender, age at disease onset, age at the time of referral to the genetic analysis, disease duration, geographical origin, and family history. The presence of the following symptoms in patients’ first-degree relatives was considered a positive family history: proximal limb muscle weakness, myotonia, and presenile cataracts. The first symptom of the disease was freely described by patients and then classified into 26 categories to allow better analysis. At the time of the first examination, the following muscle symptoms and signs were evaluated: ptosis, mastication, facial muscle weakness, speech and swallowing difficulties, sternocleidomastoid and trapezius muscle weakness, proximal and distal upper and lower limb muscle weakness, muscle reflexes, the presence of calf hypertrophy, the presence of muscle pain, back pain, muscle cramps, active and percussion hand myotonia, masseter, tongue, and facial muscles myotonia, and stiffness of the other muscle groups. Evaluation of the muscle strength in upper and lower limb muscles was assessed using the Medical Research Council (MRC) 0–5 point scale (0, no movement; 5, normal strength) [15]. To distinguish between proximal and distal muscle strength, we obtained scores for proximal limb muscles by adding individual strength of the following muscle groups on both sides: shoulder abductors and elbow flexors for upper limbs (UL) and hip flexors and knee extensors for lower limbs (LL) with a maximum score of 20. Similarly, bilateral distal limb muscle strength was calculated as a sum of wrist extensor strength for UL and foot dorsiflexor strength for LL with a maximum possible score of 10.

In 244 patients, needle electromyography (EMG) was performed. In all of them, we assessed the presence of myopathy and/or myotonia, including classical waxing-waning or less specific discharges. We also analyzed laboratory and physical evaluation directed toward multisystemic symptoms: glycemia, serum triglyceride and cholesterol levels including HDL and LDL, serum creatine kinase (CK) level, thyroid status, the presence of ophthalmological and cardiac impairments (including electrocardiography, ECG), and the presence of polyneuropathy based on a nerve conduction studies (NCS). We also assessed if a patient had frontal baldness, high arched palate, spinal deformities, extrapyramidal signs (tremor, rigor, bradykinesia), and if they used pain killers. Spirometry data and echocardiography findings were excluded from the further analysis because they were not available for at least 30% of the patients at the time of the first examination.

The following methods of descriptive statics were used: median, mean, standard deviation (SD), and proportions. Using a chi-square test, we compared the percentage of patients genetically positive for DM2, based on the presence/absence of each symptom, neurological sign, specific diagnostic findings, or any other relevant history data mentioned above. Some parameters were dichotomized based on their median values, including age at onset (before vs. after 45 years), age at sampling (before vs. after 55 years), disease duration (less vs. more than five years), proximal MRC score in UL (less vs. equal 20), distal MRC score in UL (less vs. equal 10), proximal MRC score in LL (less vs. equal 20), and distal MRC score in LL (less vs. equal 10). We also dichotomize data on glucose regulation (normal vs. impaired including glucose intolerance, insulin resistance, and diabetes mellitus), thyroid function (normal vs. hypo- or hyperthyroidism), and muscle reflexes (absent and diminished vs. normal and increased). Laboratory values were dichotomized based on the reference values of the local laboratory.

We obtained the composite score for early diagnosis of DM2. To create the *DM2 early diagnosis score* (DM2-EDS), all parameters that were associated with DM2 diagnosis at *p* < 0.01 were included in the multiple linear regression analysis (stepwise method) as independent variables, and DM2 genetic diagnosis was a dependent variable. In this way, we obtained beta values for each of the variables. Beta value shows how much dependent variable (genetic DM2 diagnosis) changes for one-unit change in an independent variable. Significant predictors obtained in the regression analysis were converted into scores based on their beta values with the least beta value marked as 1 and the total diagnostic score having a maximum value of 10. Since the least beta value that was significant (for calf hypertrophy) was 0.120, we calculated a conversion factor of 1/0.12 = 8.3. Thus, beta values for other significant variables were multiplied with 8.3 to obtain a scoring value for DM-EDS. After making the scoring system, all 291 patients were scored, and receiver operating curve (ROC) analysis was performed to test a validity of the scoring system. The area under a curve (AUC) above 0.8 was considered a good scoring system. ROC analysis allowed us to see sensitivity and specificity of individual DM2-EDS scores in making proper diagnosis of DM2. The cut-off value of DM2-EDS was selected to obtain the best sensitivity and specificity.

## Results

Genetic testing showed CCTG expansion in the *CNBP* gene confirming DM2 diagnosis in 69 (24%) of 291 patients with clinical suspicion of DM2. The median age at disease onset in the DM2 + group was 43.0 (13.0) years vs. 47.9 (16.4) years in DM2 − group (*p* = 0.02), while the median time between first symptoms and neurological examination was 9.6 (8.8) years in DM2 + group vs. 6.6 (8.4) in DM2 − group (*p* > 0.05).

**Fig. 2:**
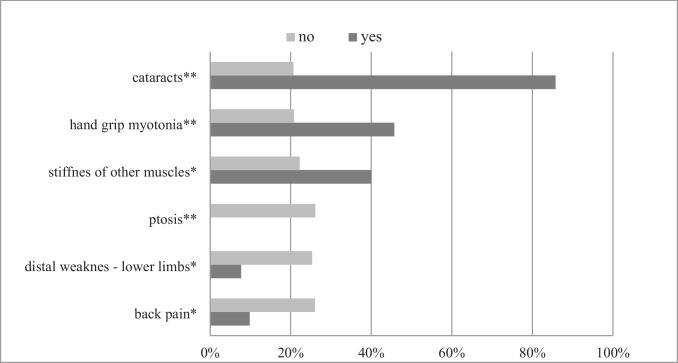
Percentage of patients with genetically confirmed DM2 based on the presence/absence of the first symptom of the disease as recalled by patients. *p < 0.05; **p < 0.01.Results are shown as percentage of genetically confirmed DM2 patients based on the presence (marked as yes) vs. absence (marked as no) of the first symptom of the disease as recalled by the patients. Only features with statistically significant difference between two groups were presented in the figure

The first symptoms significantly associated with a probability of a positive genetic test for DM2 are shown in Fig. 2. The presence of cataracts, hand grip myotonia, and other muscle myotonia was associated with the DM2 diagnosis. On the other hand, the presence of ptosis, back pain, and distal lower limb muscle weakness was suggestive of not having DM2. The following first symptoms as recalled by patients were not helpful to distinguish between DM2 + and DM2 − groups: diplopia, speech and swallowing difficulties, proximal and distal upper limb weakness, proximal lower limb weakness, myalgia, muscle cramps, tremor, fatigue, slowness, or any cardiac-related problems.

**Fig. 3:**
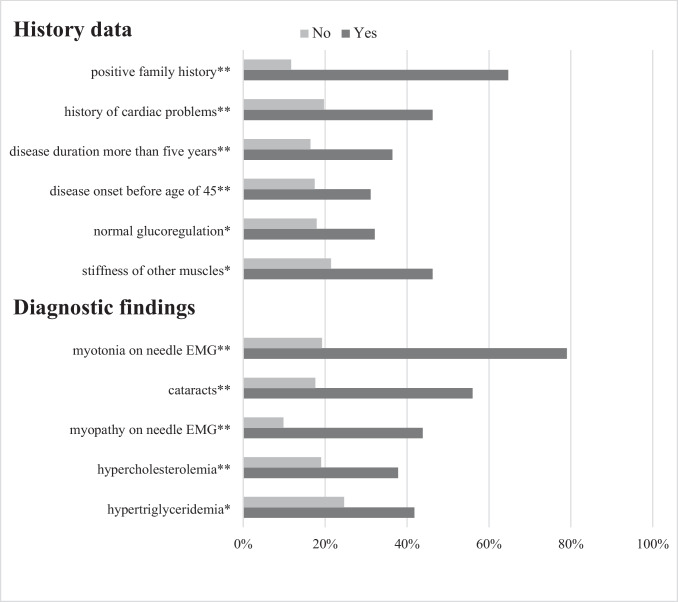
Percentage of genetically confirmed DM2 patients based on the presence/absence of history data and diagnostic findings at the time of the fir*s*t examination. *p < 0.05; **p < 0.01. Results are shown as percentage of genetically confirmed DM2 patients based on the presence (marked as yes) vs. absence (marked as no) of history data and diagnostic findings at the time of the first examination. Only features with statistically significant difference between two groups were presented in the figure

**Fig. 4:**
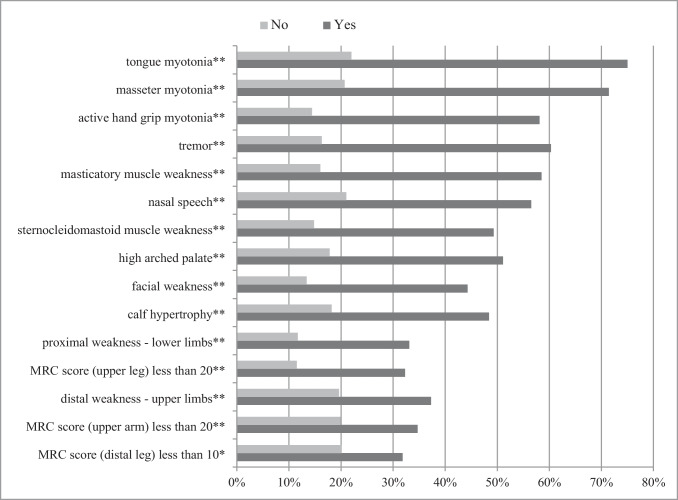
Percentage of patients with genetically confirmed DM2 based on the presence/absence of neurological signs at the time of the first examinations. *p < 0.05; **p < 0.01. MRC, Medical Research Council. Results are shown as percentage of patients with genetically confirmed DM2 based on the presence (marked as yes) vs. absence (marked as no) of neurological signs at the time of the first examination. Only features with statistically significant difference between two groups were presented in the figure

We also assessed the probability of a positive genetic DM2 diagnosis according to the presence/absence of any relevant history data, neurological signs, and additional diagnostic findings assessed at the time of the first examination. Parameters able to distinguish between DM2 + and DM2 − patients are shown in Figs. 3 and 4. All other tested parameters were not able to help in the prediction of DM2 diagnosis.

Linear regression analysis showed five different models to predict DM2 diagnosis. We selected the model with the highest adjusted *R*^2^ of 0.70, *p* < 0.001. All other models had lower *R*^2^ suggesting their inferiority to detect DM2. The following parameters appeared as significant predictors of DM2 diagnosis: cataracts (beta = 0.410, *p* < 0.001), myotonia on needle EMG (beta = 0.298, *p* < 0.001), hand tremor (beta = 0.211, *p* = 0.001), positive family history (beta = 0.171, *p* = 0.012), and calf hypertrophy (beta = 0.120, *p* = 0.043). In the final DM2-EDS, the presence of these symptoms had the following values: cataracts (present 3.4, absent 0), myotonia (present 2.5, absent 0), tremor (present 1.7, absent 0), family history (positive 1.4, negative 0), and calf hypertrophy (present 1.0, absent 0).

ROC curve of DM2-EDS for our cohort of patients is presented in figure (Online Resource 1). AUC was 0.94 (95% confidence interval of 0.90–0.97) suggesting excellent characteristics. The cut-off value of 3.25 points was associated with sensitivity of 84% and specificity of 81% to diagnose DM2, while a cut-off value of 4.6 points was associated with sensitivity of 81% and specificity of 95%.

Among 222 patients from DM2 − group, 79 (35.6%) were finally diagnosed with other diseases at our clinic (Online Resource 2). Others were not further investigated at our hospital, so we did not have access to their final diagnoses. The most common differential diagnosis in subgroup of 79 patients was a broad spectrum of other hereditary myopathies (11.7%), followed by lumbosacral radiculopathy/plexopathy (5.4%).

## Discussion

Significant predictors of the positive genetic testing for DM2 in Serbian neurology outpatients were the presence of cataracts, myotonia on needle EMG, hand tremor, positive family history, and calf hypertrophy. Early-onset posterior subcapsular cataracts is a hallmark feature of DM2, occasionally being even a presenting symptom of the disease [16–18]. In our earlier study, among 150 patients with early-onset cataracts without any neurological symptoms, 7% were genetically positive for DM2 [9]. Although early-onset cataracts is sensitive to arouse suspicion of DM2, it can be absent in these patients even in later stages of the disease. Previous studies reported frequency of lens opacities in DM2 of 32–75% [12, 19–21]. Early-onset cataracts can also be present in other systemic disorders, some of them overlapping with DM2 manifestations, like diabetes mellitus and metabolic syndrome. Diabetes mellitus, present in approximately one-third of DM2 patients [19] is, besides myopia, a leading cause of presenile cataracts [22]. Some studies also highlight the importance of the metabolic syndrome, present in more than half of DM2 patients [23], in early-onset cataracts development [24]. Nevertheless, the presence of presenile cataracts and diabetes mellitus/metabolic syndrome should guide physicians to search for subtle muscle symptoms to diagnose or exclude myotonic dystrophy.

Myotonic discharges on needle EMG are the most specific, but not mandatory electrophysiological finding in DM2 patients. Polish authors reported a frequency of electrical myotonia (EM) of 83% in DM2 patients [25]. EM is less common in DM2 compared to DM1 [26–28]. Wax and wane discharges of typical EM represent the main electrophysiological finding in DM1 patients [26]. On the contrary, myotonia in DM2 patients is frequently atypical, characterized by waning-only discharges, absent in some muscle groups or different depths of a single muscle. All of these make electrophysiological diagnosis of DM2 far more challenging than diagnosis of DM1 [26]. Atypical EM can also be seen in various neuromuscular diseases, such as muscle channelopathies, metabolic, toxic, inflammatory, and endocrine myopathies. Among these, late-onset Pompe disease (LOPD) is of specific interest. Besides EM reported in up to 76% of LOPD patients, especially in paraspinal muscles [29], LOPD and DM2 have many common symptoms, including axial and proximal limb muscle weakness, late-onset, and usually mild hyperCKemia. Thus, it is not surprising that 9% of our finally diagnosed patients from the DM2-negative group had LOPD. It is of crucial importance to single out patients with LOPD to timely introduce enzyme replacement therapy [30].

Postural hand tremor was another feature associated with a higher probability of DM2-positive genetic testing. In our earlier studies, postural hand tremor was found in 56% of DM2 patients [31], being even a presenting symptom of DM2 in 3% of them [12]. Although tremor and parkinsonism may be a feature of DM2 [32], none of these patients fulfilled the criteria for Parkinson’s disease or any atypical parkinsonism syndromes. In line with this, 58% of patients in the German DM2 cohort patients showed extrapyramidal signs and symptoms at the time of the evaluation [33]. Interestingly, one of our DM2-negative patients was eventually diagnosed with Parkinson’s disease.

Positive family history for core DM2 symptoms (proximal limb muscle weakness, presenile cataracts, and myotonia) was one of the main predictors of the CNBP gene mutation. Positive family history was previously reported in two-thirds of DM2 patients [19]. Hilbert et al. found a higher percentage of already genetically diagnosed family members in DM2 compared to DM1 patients [34]. Physicians should carefully investigate family history data in DM2 patients and even genetically screen asymptomatic first-degree relatives. It is of great importance for the detection and treatment of potentially fatal cardiac conduction defects and arrhythmias that these, although rare, may be present in DM2 patients without any other obvious symptom [20].

Calf hypertrophy is a frequent and nonspecific feature of various muscle diseases, but together with other signs, it may help to make an early diagnosis of DM2. Earlier studies reported calf hypertrophy in more than 50% of DM2 patients [17, 35]. Calf enlargement may be present in other late-onset hereditary myopathies, such as LOPD and some types of limb-girdle muscular dystrophies [36]. It has also been described in late-onset inherited motor neuron diseases, such as Kennedy disease [37], which was diagnosed in one of our DM2-suspected cases.

The final diagnosis was made in our 79 DM2-negative patients. The most common diagnosis was a broad spectrum of other hereditary myopathies. Proximal lower limb muscle weakness is present in all of them. Differential diagnosis of DM2 vs. mitochondrial myopathy can be peculiarly challenging due to a similar pattern of muscle involvement with frequent myalgias and fatigue, but also due to similar systemic manifestations: cardiac conduction abnormalities, diabetes mellitus, hypothyroidism, and gonadal failure [38]. The common differential diagnosis was lumbosacral radiculoplexopathy, presenting with painful proximal muscle weakness like in DM2. Unilateral distribution of symptoms may easily differ those entities from DM2, but the bilateral presentation is not uncommon, especially in patients with diabetic plexopathy [39]. Systemic connective tissue disorders and myositis were other diagnoses with overlapping features with DM2. Five DM2-negative patients were finally diagnosed with skeletal muscle channelopathies. Three of them had mutations in the SCN4A gene causing a paramyotonia congenita. Differential diagnosis of muscle channelopathies vs. DM2 can be challenging due to the occasional presence of muscle pain and weakness in channelopathies [40]. Three of our DM2-negative patients were finally diagnosed with myasthenia gravis. Proximal muscle weakness and fatigue are frequently reported in both myasthenia and DM2 patients. In line with this, Hilbert and colleagues reported DM2-positive patient, diagnosed 6 years after the initial misdiagnosis of myasthenia gravis [34]. Multiple sclerosis, diagnosed in one of our DM2-negative patients, is another possible differential diagnosis to DM2, primarily due to increased muscle reflexes and white matter hyperintensities in the brain seen in DM2 patients [41].

The main limitation of our study is that it was conducted in a tertiary university hospital in country where prevalence of DM2 seems to be among the highest in Europe [42]. Thus, our starting point seems to include a bias toward a selected sample. However, we believe the final results with the scoring system that included very simple predictors could be used in other clinical settings and in general population. Of course, this should be further confirmed in validation studies not only in Serbia and other countries of Central and Eastern Europe where DM2 is more common, but also in other regions.

## Conclusion

Significant predictors of DM2 diagnosis in the neurology outpatient unit were identified. We made an easy-to-administer DM2-EDS score for early diagnosis of DM2.

## Supplementary information

Below is the link to the electronic supplementary material.

Supplementary file1 (PDF 258 KB)

Supplementary file2 (PDF 272 KB)

## Data Availability

Raw data were generated at Neurology Clinic, Clinical Center of Serbia, Faculty of Medicine, University of Belgrade, Belgrade, Serbia. The data that support the findings of this study are available from the corresponding author upon reasonable request.
