# Supplementary material for: Clinical score for early diagnosis of myotonic dystrophy type 2
Source: Neurol Sci. 2022 Nov 19;44(3):1059–67. doi: 10.1007/s10072-022-06507-9 (PMC9925479; doi:10.1007/s10072-022-06507-9)
Supplement: Supplementary file 1 — Supplementary file1 (PDF 258 KB) [file 10072_2022_6507_MOESM1_ESM.pdf]

Clinical score for early diagnosis of myotonic dystrophy type 2

Neurological Sciences

Vukan Ivanovic[1], Stojan Peric[1]*, Jovan Pesovic[2], Radoje Tubic[3], Ivo Bozovic[1], Ivana Petrovic[4], Dusanka Savic-Pavicevic[2], Giovanni Meola[5], Vidosava Rakocevic-Stojanovic[1]

[1]University of Belgrade – Faculty of Medicine, University Clinical Center of Serbia – Neurology Clinic, Belgrade, Serbia

[2]University of Belgrade – Faculty of Biology, Center for Human Molecular Genetics, Belgrade, Serbia

[3]Institute of Oncology and Radiology of Serbia, Belgrade, Serbia

[4]University of Belgrade – Faculty of Medicine, University Clinical Center of Serbia – Endocrinology Clinic, Belgrade, Serbia

[5] Department of Neurorehabilitation Sciences - Casa di Cura del Policlinico, Department of Biomedical Sciences for Health, University of Milan, Italy

* Corresponding Author:

Stojan Peric, MD, PhD, neurologist

Email. stojanperic@gmail.com

Online Resource 1. ROC curve analysis of DM2-EDS in our cohort of patients

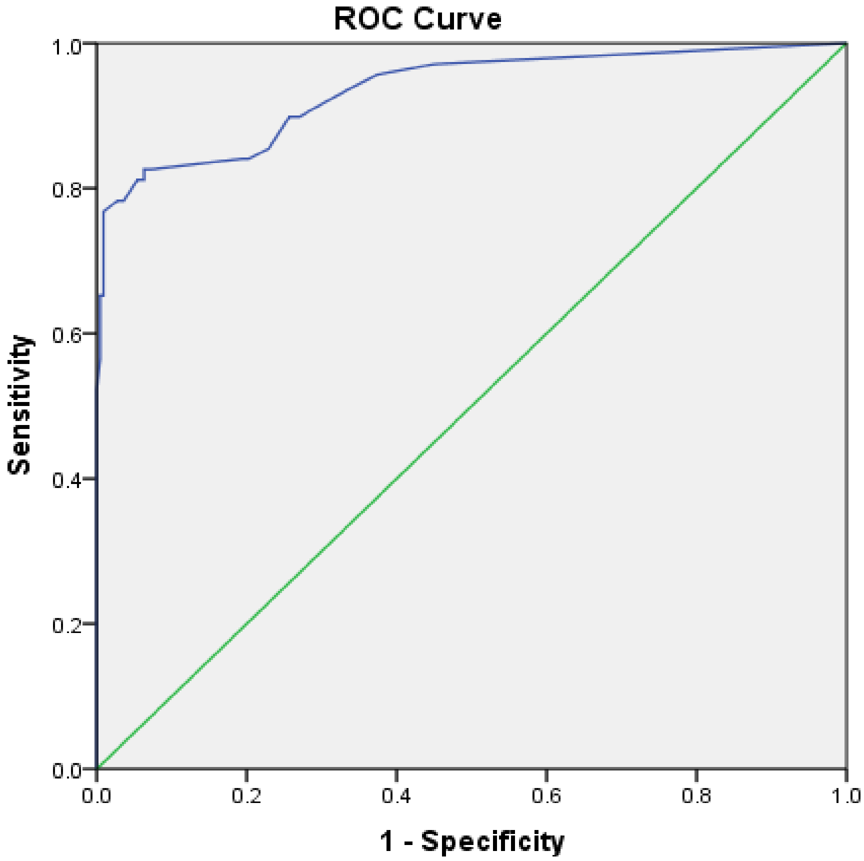

DM2-EDS – DM2 Early Diagnosis Score; ROC – Receiver operating characteristic
