# Supplementary material for: Clinical score for early diagnosis of myotonic dystrophy type 2
Source: Neurol Sci. 2022 Nov 19;44(3):1059–67. doi: 10.1007/s10072-022-06507-9 (PMC9925479; doi:10.1007/s10072-022-06507-9)
Supplement: Supplementary file 2 — Supplementary file2 (PDF 272 KB) [file 10072_2022_6507_MOESM2_ESM.pdf]

**Clinical score for early diagnosis of myotonic dystrophy type 2**

**Neurological sciences**

Vukan Ivanovic[1], Stojan Peric[1]*, Jovan Pesovic[2], Radoje Tubic[3], Ivo Bozovic[1], Ivana Petrovic[4], Dusanka Savic-Pavicevic[2], Giovanni Meola[5], Vidosava Rakocevic-Stojanovic[1]

[1]University of Belgrade – Faculty of Medicine, University Clinical Center of Serbia – Neurology Clinic, Belgrade, Serbia

[2]University of Belgrade – Faculty of Biology, Center for Human Molecular Genetics, Belgrade, Serbia

[3]Institute of Oncology and Radiology of Serbia, Belgrade, Serbia

[4]University of Belgrade – Faculty of Medicine, University Clinical Center of Serbia – Endocrinology Clinic, Belgrade, Serbia

[5] Department of Neurorehabilitation Sciences - Casa di Cura del Policlinico, Department of Biomedical Sciences for Health, University of Milan, Italy

* Corresponding Author:

Stojan Peric, MD, PhD, neurologist

Email. stojanperic@gmail.com

Online Resource 2. Final diagnosis obtained at our clinic in patients genetically negative for DM2 (n=79)

| Final diagnosis | N | Final diagnosis | N |
|---|---|---|---|
| **Hereditary myopathies** | **26 (33%)** | **Fatigue and myalgic syndromes** | **5 (6.3%)** |
| Limb-girdle muscular dystrophy[a] | 7 | Chronic fatigue syndrome | 2 |
| Pompe disease | 4 | Fibromyalgia | 2 |
| Mitochondrial myopathy | 4 | Polymyalgia rheumatica | 1 |
| Facioscapulohumeral muscular dystrophy | 3 | | |
| Oculopharyngeal muscular dystrophy | 2 | **Acquired myopathies** | **5 (6.3%)** |
| NEB-related myopathy[b] | 1 | Vitamin D deficiency | 1 |
| MYH6-related myopathy[c] | 1 | Corticosteroid-induced myopathy | 1 |
| MYH7-related myopathy[d] | 1 | Alcoholic myopathy | 1 |
| FLNC-related myopathy[e] | 1 | Drug-induced myopathy | 1 |
| CAV3-related distal myopathy[f] | 1 | Hypothyroid myopathy | 1 |
| Genetically non-defined congenital myopathy | 1 | | |
| | | **Channelopathies** | **5 (6.3%)** |
| **Lumbosacral radiculopathy/plexopathy** | **12 (15.2%)** | SCN4A channelopathy | 3 |
| Radiculopathy with or without spinal stenosis | 9 | CLCN1 channelopathy | 1 |
| Diabetic plexopathy | 3 | Genetically undefined channelopathy | 1 |
| | | | |
| **Systemic connective tissue disorders** | **7 (8.9%)** | **Myositis** | **4 (5.1%)** |
| Non-specified | 5 | Idiopathic myositis | 3 |
| Still disease | 1 | Virus myositis | 1 |
| Systemic vasculitis | 1 | | |
| | | **Neuromuscular junction disorders** | **4 (5.1%)** |
| **Other neuromuscular disorders** | **7 (8.9%)** | Myasthenia gravis | 3 |
| Asymptomatic hyperCKaemia | 2 | Lambert-Eaton myasthenic syndrome | 1 |
| Paraproteinemic neuropathy | 2 | | |
| Late-onset nemaline myopathy | 1 | **Other neurological disorders** | **4 (5.1%)** |
| Pure motor CIDP[g] | 1 | Dystonia | 2 |
| Kennedy disease | 1 | Parkinson disease | 1 |
| | | Multiple sclerosis | 1 |

[a]dysferlinopathy (n=2), TRIM32-related myopathy (n=2), calpainopathy (n=1), LAMA2 myopathy (n=1), genetically non-defined limb-girdle muscular dystrophy (n=1); [b]NEB – nebulin; [c]MYH6 – myosin heave chain α; [d]MYH 7 – myosin heavy chain β; [e]FLNC – filamin C, [f]CAV3 – caveolin 3; [g]CIDP – chronic inflammatory demyelinating polyradiculoneuropathy
